# A dynamic *in vivo*-like organotypic blood-brain barrier model to probe metastatic brain tumors

**DOI:** 10.1038/srep36670

**Published:** 2016-11-10

**Authors:** Hui Xu, Zhongyu Li, Yue Yu, Saman Sizdahkhani, Winson S. Ho, Fangchao Yin, Li Wang, Guoli Zhu, Min Zhang, Lei Jiang, Zhengping Zhuang, Jianhua Qin

**Affiliations:** 1 Division of Biotechnology, Dalian Institute of Chemical Physics, Chinese Academy of Sciences, Dalian, China; 2 University of Chinese Academy of Sciences, Beijing, China; 3 Surgical Neurology Branch, National Institute of Neurological Disorders and Stroke, National Institutes of Health, Bethesda, Maryland, USA

## Abstract

The blood-brain barrier (BBB) restricts the uptake of many neuro-therapeutic molecules, presenting a formidable hurdle to drug development in brain diseases. We proposed a new and dynamic *in vivo*-like three-dimensional microfluidic system that replicates the key structural, functional and mechanical properties of the blood-brain barrier *in vivo*. Multiple factors in this system work synergistically to accentuate BBB-specific attributes–permitting the analysis of complex organ-level responses in both normal and pathological microenvironments in brain tumors. The complex BBB microenvironment is reproduced in this system via physical cell-cell interaction, vascular mechanical cues and cell migration. This model possesses the unique capability to examine brain metastasis of human lung, breast and melanoma cells and their therapeutic responses to chemotherapy. The results suggest that the interactions between cancer cells and astrocytes in BBB microenvironment might affect the ability of malignant brain tumors to traverse between brain and vascular compartments. Furthermore, quantification of spatially resolved barrier functions exists within a single assay, providing a versatile and valuable platform for pharmaceutical development, drug testing and neuroscientific research.

Drug development for central nervous system (CNS) diseases is particularly challenging due to the limited ability of existing models to emulate the properties of the blood-brain barrier (BBB)^1^. The BBB is a selective yet dynamic barrier between the circulatory system and the CNS, which is formed by microvascular endothelial cells (BMECs) that line cerebral capillaries, pericytes and perivascular end-feet of astrocytes (Fig. 1a,b). It plays a central role in maintaining homeostasis of the CNS interstitial fluid that normal neuronal functions depend on^2^,^3^. BBB dysfunction is implicated in a range of pathologic conditions such as neurodegenerative disorders, stroke, infection and brain tumors^4^,^5^,^6^,^7^. Reproducing the physiological characteristics and the functional responses of the BBB in a reliable model will greatly accelerate the development of novel therapeutics for CNS diseases. However, establishing a robust system that recapitulates the complex BBB physiology and cytoarchitecture remains an elusive goal.

BBB studies have depended on *in vivo* animal^8^,^9^ and *in vitro* cell-based models^10^,^11^,^12^. *In vivo* systems provide the benefit of studying the BBB in its natural milieu, but restrict to understand the mechanisms that govern normal physiological processes or pathologic conditions. It is also impossible to perform quantitative studies or high throughput assay *in vivo*. In contrast, BBB models *in vitro* utilize cultured cells types, such as BMECs, astrocytes or pericytes on two-sides of a coated polycarbonate Transwell plate and are popular due to their simplicity^10^,^11^,^12^,^13^,^14^,^15^,^16^. However, these simplified models lack the dynamic mechanical microenvironment and complex architecture of the BBB. A fiber-based approach(DIV-BBB) has been proposed to mimic the BBB with dynamic flow, but the cumbersome design precludes scalability and does not allow for visualization of changes in vascular endothelium morphology^17^,^18^,^19^,^20^,^21^,^22^. Advances in micro-scale engineering technologies have recently made it possible to create microfluidic devices that are lined with living cells to mimic the micro-architecture of an organ *in vitro*, allowing to build a model of BBB on a microdevice^23^,^24^,^25^,^26^,^27^,^28^,^29^,^30^. These microscale BBB models are commonly achieved by fabricating a sandwich chip lined with living cells on the two sides of a polycarbonate membrane, resembling the traditional Transwell system. However, the obtained barrier functions and assay throughput are very limited. To our knowledge, such technology has thus far been unable to provide a BBB model that incorporates multiple physiologic parameters and re-creates the cytoarchitecture *in vivo* that may also be amenable to potential high throughput assay.

Here, we developed a novel microfluidic system that can effectively replicate the complex multicellular architecture, mechanical properties, 3D extracellular matrix (ECM) and functional responses of the blood-brain barrier in normal and pathologic conditions. We investigated the contributions of vascular flow, and direct co-culturing of endothelial cells and astrocytes on 3D ECM to the integrity function of the BBB *in vitro*. We explored the unique capability of this robust system for examination of brain metastasis and the therapeutic response of brain tumors in the context of the complex BBB microenvironment. This system carries promise to be a powerful platform for the study of BBB physiology and for effective evaluation of CNS therapeutics.

## Results

### Microdevice design and operation

**Figure 1:**
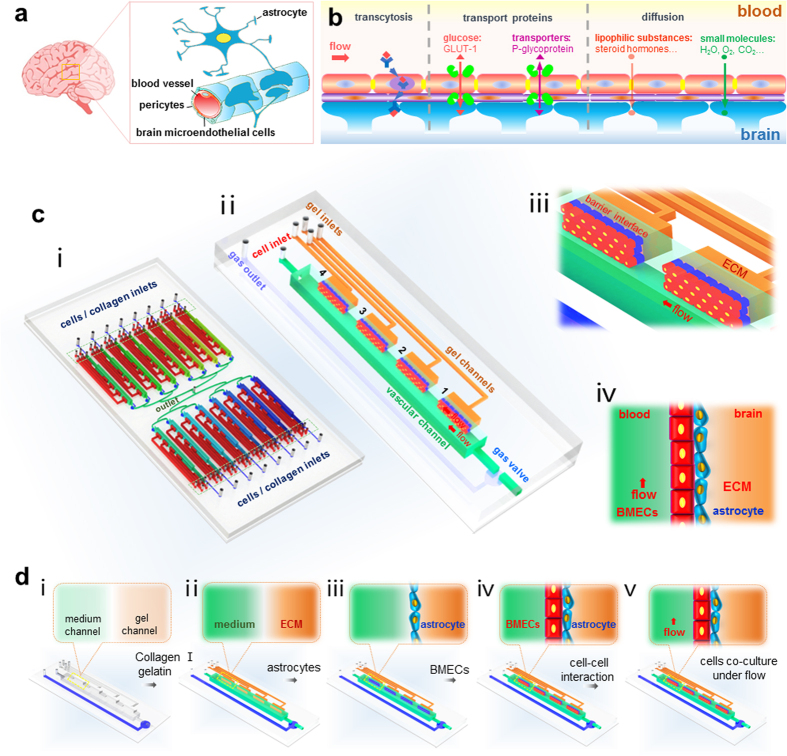
The integrity and function of blood-brain barrier. (**a**) Cellular constituents of the BBB *in vivo*. The BBB is formed by lined BMECs surrounded by pericytes and astrocytic end-feet. (**b**) Schematic illustration of BBB function with the expression of several transporters and functional proteins. (**c**) The design and structure of the integrated BBB device. (i) Device design. It is composed of 16 independent function units connected by a microchannel network (ii). Each unit consists of four uniform BBB regions, one vascular channel, one gas channel, one gas valve and four gel channels. They share the same waste outlet in the middle of the chip. Enlarged view (iii) and sideview (iv) of the barrier regions consisting of BMECs, astrocytes and 3D ECM under flow. (**d**) Illustration of the procedures to establish the blood-brain barrier under flow conditions. (i) The empty device with gas valve and vascular channels closed. (ii) Collagen gelatin and cell medium infusion with gas valve opened. (iii) Suspension of astrocytes perfused into the vascular channel and attached to the side surface of gelled ECM. (iv) Suspension of BMECs perfused into the vascular channel and attached to the astrocytes. (v) Co-cultures of BMECs and astrocytes in the vascular channels under continuous flow.

We designed a 3D microfluidic device that reproduces the key structural, functional and mechanical properties of the blood-brain barrier *in vitro*. This was accomplished by fabricating a poly-dimethylsiloxane (PDMS) microfluidic device containing an array of 16 independent functional units connected by a micro-channel network (Fig. 1c). Each functional unit consists of four uniform blood-brain barrier regions. Each region consists of one vascular channel for introducing fluidic flow (vascular compartment) and one channel for infusing natural extracellular matrix (ECM) collagen or astrocytes (brain compartment) (Fig. 1c i–iv). The compartmentalized channel configuration of the microdevice makes it possible to manipulate vascular flow and to deliver cells and nutrients to the vascular or brain compartments independently. The parallel design of functional units facilitates the BBB assay in a high throughput manner.

To establish the functional blood-brain barrier, primary rat BMECs and astrocytes were first extracted and characterized (Supplementary Figs 1 and 2). The steps to establish the dynamic barrier are illustrated in Fig. 1d. Collagen gels, primary astrocytes and BMECs were sequentially infused into the microdevice via the respective channels. Once astrocytes were infused and grown to confluence on 3D ECM gels, endothelial cells were introduced and allowed to attach to the astrocytes forming a continuous cell layer on the ECM with direct cell-cell contact. The co-cultured cells were exposed to a controlled low flow through the vascular channel (0.1 dyne/cm^2^)^31^, mimicking the capillary flow in the brain. Under this condition, the BMECs were grown to form intact barrier in the dynamic co-culture with astrocytes on the surface of 3D ECM after 48 h. These cells remained viable for one week after flow was introduced into the vascular microchannels.

To operate the dynamic BBB model system, the 16 addressable functional units of the microdevice were individually monitored with an air pressure pump to introduce fluidic flow, brain cells or drug compounds in parallel (Supplementary Fig. 3a,b). Time-lapse images of each functional unit were captured by real-time fluorescent microscopy. Supplementary Fig. 3c shows the fluorescent images of 64 barrier regions with BMECs stained with red and astrocytes with blue. Supplementary Fig. 4 shows the 3D confocal structures of one barrier region consisting of BMECs and astrocytes on ECM. It appears the two types of cells are interacted and fused together closely, which are obviously different from the indirect interaction commonly observed using traditional Transwell assay. This system design creates a physiologically relevant BBB model of the endothelial- astrocyte layer on ECM with the ability to simulate vascular flow and to perform multiple experiments in parallel.

### Physiological changes in barrier integrity and function

**Figure 2:**
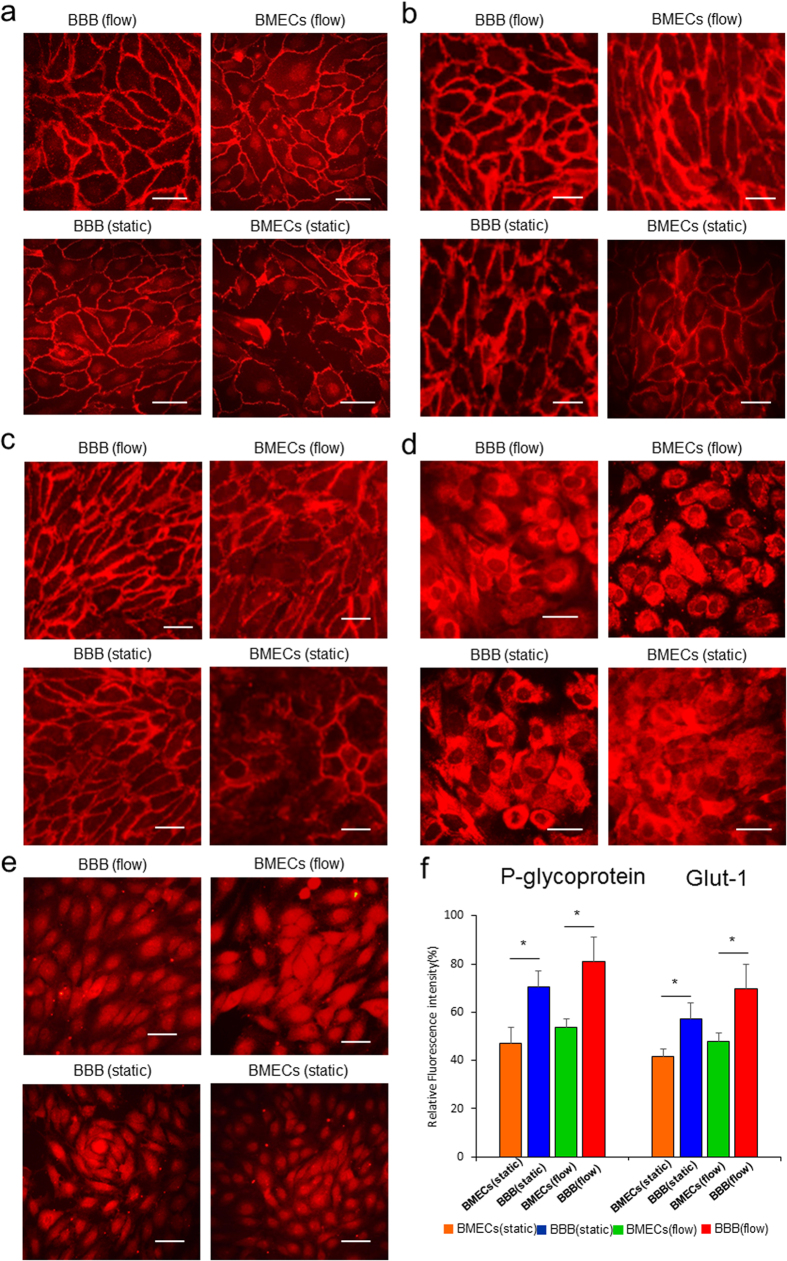
Expression and quantification of the barrier-specific functional proteins in BMECs under both static and flow conditions after 48 hours. **(a–c)** Expression of adhesive protein VE-cadherin (**a**), tight junction proteinZO-1 (**b**) and claudin-5 (**c**). n = 3. **(d,e)** Expression of efflux transporter of P-glycoprotein (**d**) and glucose transporter Glut-1 (**e**) in BMECs with or without the presence of astrocytes. The flow rate applied to the vascular channel is 1 μL/min, and all scale bars indicate 50 μm. (**f**) Relative fluorescence intensity statistics of the expression of P-glycoprotein and Glut-1 in different groups. n = 3. Data are presented as mean ± s.e.m. Statistical significance was calculated by Student t-test. *P < 0.05.

Endothelial cells of BBB express specific features that contribute to its barrier properties, including expression of tight junction proteins, influx glucose transporter (Glut-1) and efflux transporter (P-gp/MDR1)^32^,^33^. We examined how the presence of dynamic flow or astrocytes co-culture alter the expression of these proteins in endothelial cells by using immunofluorescence assay (Fig. 2a–f). Exposure to dynamic flow (1 μL/min) significantly increased expression of endothelial tight junction proteins (ZO-1, Claudin-5) and adhesive protein (VE-Cadherin) in BMECs. Moreover, the addition of astrocytes to BMECs also increased the expression of these three proteins when compared to BMECs alone. This result suggests that both astrocytes and vascular flow would contribute to the maintenance of barrier integrity by enhancing expression of tight junction proteins in endothelial cells. Furthermore, we examined the expression of transporters as P-gp and Glut-1 in BMECs in similar conditions. We found that the presence of astrocytes increased the expression of P-gp and Glut-1 under both static and dynamic flow conditions. However, the presence of flow alone did not alter the expression of these transporters, suggesting that astrocytes play a critical role in modulating expression of these proteins (Fig. 2d–f).

**Figure 3:**
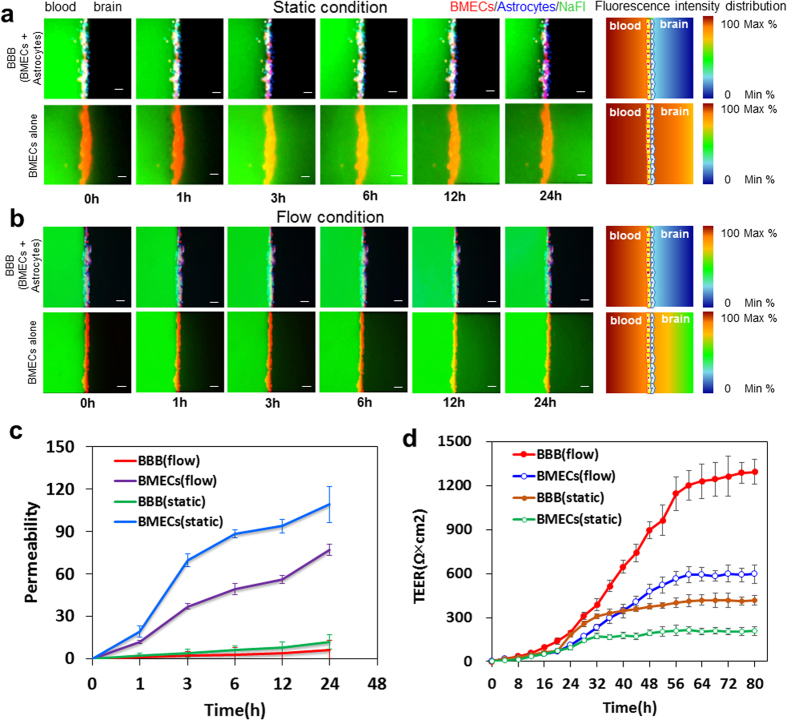
Evaluation of the barrier function of the 3D high throughput BBB system. (**a,b**) Time-lapse images of permeable sodium fluorescein tracer (NaFl, MW = 376Da, 250 μM) across the BBB into the brain compartment under the static (**a**) and flow condition (**b**) over 24 h. Green, NaFl. Scale bar, 25 μm. (**c**) Quantitative graphs showing the permeability of NaFl across the BBB into the brain compartment in BBB and BMECs alone under the static and flow conditions. (**d**) TEER measurement of the barrier function in the BBB group and BMECs alone under static and flow conditions. The maximal value of TEER in the BBB group under flow was 1298 ± 86 Ω × cm^2^. n = 3. Data are presented as mean ± s.e.m.

The BBB model *in vivo* is also characterized by its impermeability to diffusion of small polar molecules. Using a low-molecular-weight hydrophilic sodium fluorescein tracer (NaFl, 376 Da), we examined the permeability of the blood-brain barrier in our model to small hydrophilic molecules. Fig. 3a,b shows the time-lapse images of fluorescein diffusion through the BBB under static and dynamic conditions with or without astrocytes in co-culture. Consistent with previous results, diffusion of fluorescein is significantly diminished by the presence of astrocytes under both static and dynamic conditions. The presence of dynamic flow was able to diminish the permeability of BMECs layer without astrocytes, but it did not further enhance the impermeability in the presence of astrocytes (Fig. 3c). Taken together, these results suggest that the presence of astrocytes enhances the impermeability of the BBB against small hydrophilic molecules, a known property of BBB *in vivo* that is recapitulated in our model.

To further assess the endothelial barrier integrity in this system, we measured the transendothelial electrical resistance (TEER) across the blood-brain barrier over the course of four days (Fig. 3d). TEER is a widely used parameter to characterize and evaluate the integrity of the tight junction of the barrier of endothelial and epithelial cell monolayers. Measuring TEER across the barrier could provide real-time information on barrier quality. Therefore, it is an ideal method to monitor the barrier function in blood-brain barrier system. Consistent with above findings, we found that the presence of dynamic flow and astrocytes both increased TEER of BMECs significantly. The presence of dynamic flow increased the TEER of BMECs mono-culture by more than 4-fold. The addition of astrocytes, in the presence of dynamic flow, further enhanced the TEER up to a maximum value of 1298 ± 86 Ω × cm^2^. Such value far exceeded the reported TEER for Transwell-based BBB models^10^,^11^,^12^,^13^,^14^,^15^,^16^. The exceptionally high TEER represents the formation of a more stringent and selective vascular structure in this dynamic 3D BBB system. Furthermore, we noticed that the TEER reached a steady-state within 60 h (<3 days), which is consistent with the minimum time required for obtaining barrier integrity and low molecular permeability as measured above.

### Modeling of extravasation in brain metastasis

**Figure 4:**
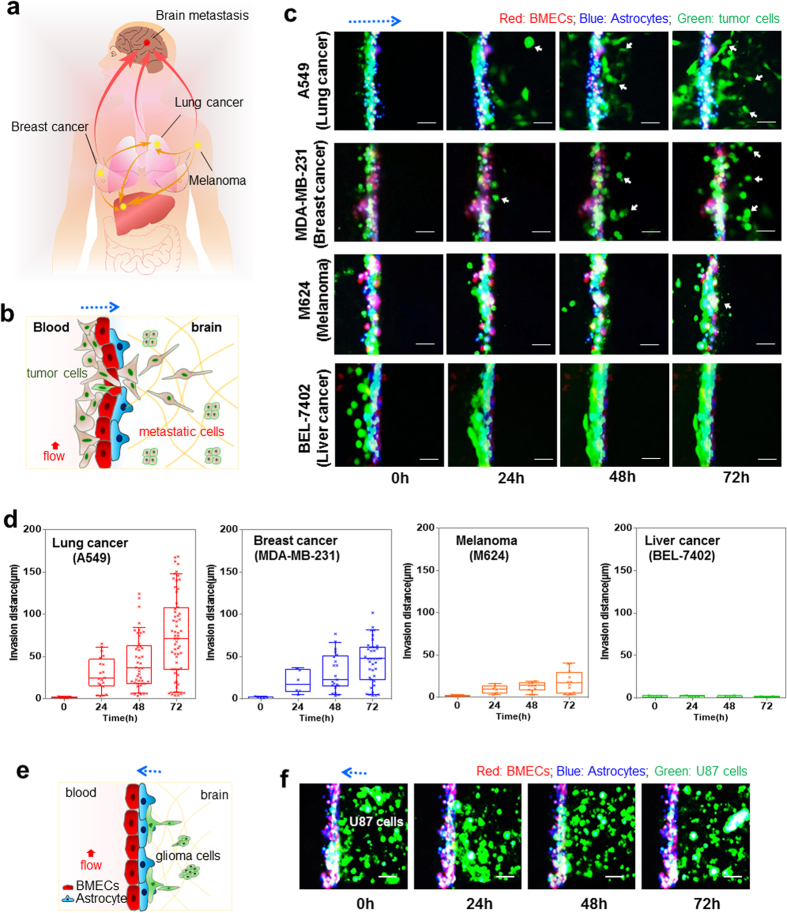
Brain metastasis of malignant cancer cells. (**a**) Schematic illustration of the brain metastasis of the common cancer cells into the brain, such as lung cancer, breast cancer and melanoma. Metastases from primary sites spread to the brain through the circulatory system (red arrows) and also to adjacent sites (orange arrows). (**b**) Schematic of exogenous cancer cells penetrating the brain by crossing the BBB. (**c**) Time-lapse images of extravasation of different cancer cells across the barrier on this BBB system in lung cancer cells (A549), breast cancer cells (MDA-MB-231), melanoma (M624) and liver cancer cells (BEL-7402). The migration of cancer cells across the BBB was monitored over 72 h. The flow rate is 1 μL/min. The cells were pre-labelled with live-cell staining dyes. Red, BMECs; Blue, astrocytes; Green, cancer cells. Arrows, metastatic cancer cells into the brain compartment by crossing the BBB. (**d**) Box-and-whiskers plots of cell migration of different cancer cells crossing the BBB. The box represents the 25^th^ and 75^th^ percentiles with the median shown by the line bisecting the box. Invasion distance is shown by crosses inside the box. The whiskers represent the 10^th^ and 90^th^ percentiles of the data. (**e**) Time-lapse migration of glioma U87 cells in the brain compartment under vascular flow. U87 cells could not traverse the BBB into the vascular compartment after 72 h. Flow rate, 1 μL/min. Red, BMECs; Blue, astrocytes; Green, U87 cells. Scale bar, 100 μm.

Beyond mimicking BBB physiology and function, we further explored the potential value of this system to replicate more complex disease processes such as brain metastases. It is well known that specific cancers, such as lung, breast and melanoma, have a greater propensity to metastasize to the brain than others in animal models (Fig. 4a,b)^34^,^35^. To form brain metastasis, tumor cells must cross the BBB into the brain^36^. Here, we explored the capability of this system to reproduce the process of malignant cell extravasation across the BBB by infusing various cancer cell types through the vascular compartment. Fig. 4c shows the time-lapse images of four different cancer cell types, pre-dyed to green, crossing the BBB over 72 hours using fluorescent imaging. The individual images of different cell types lined in BBB were shown in Supplementary Fig. 5, demonstrating the localization of each cell type. These results demonstrated the inter-play between the cancer cells and BBB. It appeared the integrity of BBB was disrupted by migrated lung cancer cells (A549), breast cancer cells (MDA-MB-231) and melanoma cells (M624), while not by liver cancer cells (BEL-7402). These results demonstrated the inter-play between cancer cells and endothelial barrier of the BBB. It recapitulated the clinical finding that lung cancer, breast cancer and melanoma cells have the ability to cross the BBB while others like liver cancer do not. To quantify the invasion assay, we plotted the migration distance as a function of time (Fig. 4d). Liver cancer cell line BEL-7402 did not demonstrate any migration across the BBB over 72 hours, while lung cancer (A549), breast cancer (MDA-MB-231) and melanoma (M624) cells showed various degrees of migration over the same period. Conversely, we also investigated the ability of brain tumor cells to traverse the BBB from the brain compartment to the vascular compartment. U87 glioma cells, derived from highly invasive brain tumor, were seeded in the 3D collagen gel of the brain compartment. Despite its inherent aggressiveness, U87 cells were unable to cross the BBB (Fig. 4e,f). This result, again, recapitulated the clinical finding that despite its aggressive nature, glioma almost never metastasizes out of the cerebral spinal fluidic (CSF) space.

The process of brain metastases was further explored by investigating co-cultured cancer cell lines with astrocytes, the most abundant cells type in the brain. When U87 cells were plated with astrocytes, they intermixed and formed a homogenous cell layer within 72 hours. However, when A549, MDA-MB-231 or M624 cells were co-cultured with astrocytes, the cancer cells did not integrate with astrocytes but rather self-segregated and formed cell spheres (Supplementary Fig. 6). Taken together, these results suggest that the possible interactions between cancer cells and astrocytes in BBB might affect the ability of the malignant tumor cells to traverse between brain tissue and vascular compartments. This system provides a unique platform to study this metastatic process in a high resolution and high throughput manner.

### Evaluation of therapeutic response in glioma brain tumor

**Figure 5:**
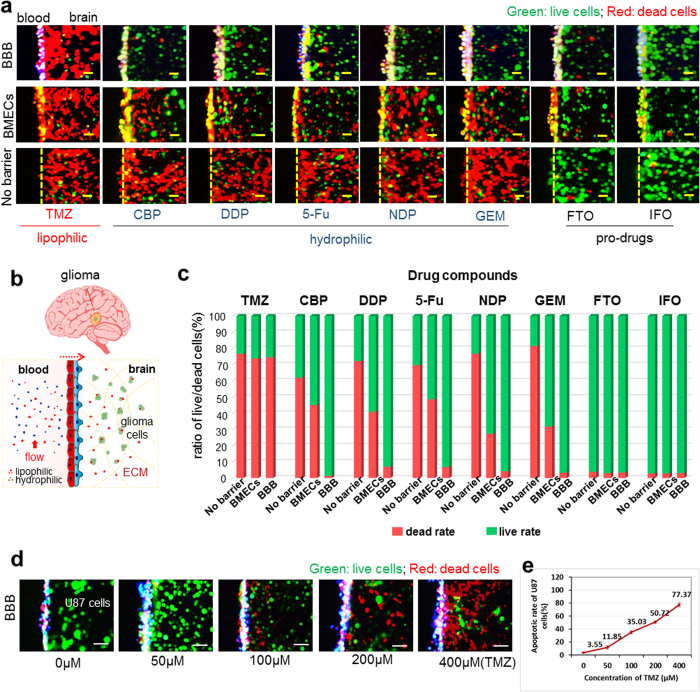
Therapeutic response of glioma cells to clinically relevant pharmaceutical agents on this BBB system. (**a**) Characterization of U87 glioma cells in various barrier groups after the addition of eight different chemotherapeutic agents on the vascular compartment under flow. TMZ: lipophilic molecule. CBP, DDP, 5-Fu, NDP and GEM are hydrophilic molecules. FTO and IFO are pro-drugs with hydrophilic properties. The U87 cells were labelled with live/dead staining dyes. Red: dead cells; Green: live cells. (**b**) Schematic of diffusion of lipophilic and hydrophilic drug compounds across the BBB, and (**c**) Quantitative assay of live/dead rate of U87 cells triggered by the drugs introduced into the vascular compartment of the BBB. n = 3. (**d**) The functional response of the BBB to lipophilic TMZ penetration at different concentrations. The U87 cells exhibited dose-dependent responses to the TMZ added in the vascular compartment. Green, live cells; Red, dead cells. Scale bar, 25 μm. (**e**) Plot of the rate of apoptotic U87 cells as a function of TMZ concentration. n = 3.

The BBB represents a unique challenge of drug delivery to the brain. The lack of a reliable model to predict BBB penetration has impeded the development of CNS therapeutics. We explored whether our platform could be used as a screening tool for drug development for CNS diseases. As a proof of concept, we used our model to compare the efficacy of various known compounds in treatment for brain tumors (Fig. 5a,b). We created a complex brain tumor microenvironment by incorporating the blood-brain barrier, dynamic vascular flow and brain tumor cells within the 3D ECM. Pharmaceutical agents widely used in clinical with well-known properties were introduced to the vascular channels under flow conditions. Eight clinically relevant chemotherapeutic agents were tested on their ability to cross the BBB and their cytotoxic effects on glioma cells. The properties of these agents are listed in Supplementary Table 1^37^,^38^,^39^,^40^,^41^,^42^,^43^,^44^. Among them, only Temozolomide (TMZ) is lipophilic and permeable to BBB, which is used for the treatment of glioblastoma multiforme in clinics. The other seven compounds are hydrophilic and broad-spectrum anti-cancer agents with poor BBB permeability. We choose these drugs with different properties to evaluate the permeability of the established barrier against these pharmaceuticals.

In order to evaluate the drug with a higher anti-tumor efficacy, we adopted the dose as twice as their IC_50_ without extensive optimization for the drug testing. Fig. 5c compares the ability of each compound to induce apoptosis in U87 glioma cells under three conditions – no barrier, barrier with only BMECs or barrier with co-cultured BMECs and astrocytes. The results showed that only TMZ could significantly induce apoptosis in glioma cells (labelled with ethidium homodimer-1) when BBB was present. The other five compounds (CBP, DDP, 5-Fu, NDP and GEM) were able to induce apoptosis in the absence of BBB, but not when the barrier was present. This demonstrated that while these compounds themselves had cytotoxicity against U87 cells, their inability to cross the BBB render them ineffectual against CNS pathology. FTO and IFO had no activity against U87 with or without BBB. This is an expected outcome since these are pro-drugs, which must be metabolized by the liver to generate the active compounds. In principle, FTO and IFO could not take effects without metabolized by liver cells, thus, we can compare the efficacy of anticancer drugs against glioma cells in this complex BBB model system using pro-drugs as a control. By testing these drugs with different properties (solubility, permeability to BBB, drug metabolism process et al) on the device, we can not only evaluate the selective permeability of the established barrier against anti-cancer drugs, but also assess their efficacy to induce apoptosis of cancer cells in a complex brain tumor microenvironment. We also investigated the dose responses of U87 cells to TMZ (50–400 μM). Fig. 5d,e depicted the percentage of apoptotic cells increased in a dose-dependent manner after 48 hours of drug administration. At 400 μM, TMZ was able to induce apoptosis in over 77% of U87 cells. Taken together, these results demonstrate the utility and reliability of this model in evaluating drug penetration of the BBB. Combined with the ability to perform parallel experiments, this platform could be effectively used for high throughput screening for CSF penetrating compounds for various CNS pathologies.

## Discussion

This biomimetic microsystem offers many unique capabilities that provide added value beyond those available in current *in vitro* BBB or preclinical animal models. With this robust BBB system, we were able to independently monitor and flexibly adjust all system parameters, including the presence or absence of different cell types (e.g. endothelial cells, astrocytes), accurate flow rate and 3D ECM components, while simultaneously analyzing the organ-level BBB responses in real time with spatio-temporal resolution (Supplementary Table 2).

It is noteworthy, we obtained a higher TEER value around 1300 Ω × cm^2^, far exceeding the reported TEER in Transwell-based BBB models^10^,^11^,^12^,^13^,^14^,^15^,^16^. The high TEER represents the formation of a more stringent and selective vascular structure in this dynamic BBB system. We presume that several factors might attribute to the higher TEER obtained in our BBB model. (1)The barrier was reconstructed based on co-cultured BMECs and astrocytes on 3D ECM gels, analogous to extracellular microenvironment of brain; (2) Co-cultured BMECs and astrocytes with direct contact may promote the intercellular interactions as existing *in vivo*. (3) The addition of flow condition recapitulates the mechanical cues happened in vascular network.

In particular, the unique capability to reproduce the time-scale extravasation of exogenous cancer cells in brain metastasis facilitates our understanding of the interplay of the endothelial barrier function and metastatic cancer cells. This system can also be applied to simulate the biological barriers required for metastasis in other tissues or organs. The ability to assess brain tumor response to therapy within the specific microenvironment in a high-throughput manner is the first of its kind. Also, with the capacity to microdissect and analyze cells pre- and post- treatment, we are able to investigate the tumor cellular and microenvironmental changes as it relates to the endothelial barrier. Meanwhile, this model could be extended for the study of mechanism involving complex cell-cell, cell-matrix and cell-signaling factors interactions in 3D tumor microenvironment. It is also feasible to deeply explore the signaling factors that can promote cells sprouting and angiogenesis. This model may also be applied to inflammatory and degenerative neurological conditions, such as Alzheimer’s and Parkinson’s disease. In fact, any neurological disorder with a cellular model may be utilized in this BBB system. Thus it offers a powerful platform for the investigation of complex patho-physiology of the BBB in various disease processes and enables more rapid, accurate, cost-effective and drug testing. Particularly, it has the potential to integrate with other organ-on-a-chip devices to accelerate the development of novel clinical strategies and pharmacological treatments to reduce the burden of many CNS diseases and precision medicine.

Great efforts have been made to establish experimental models to reflect the blood brain barrier. Currently, both *in vitro* designs have attempted to recreate the blood brain barrier on a sandwich microchip, but lacks the biological capability in recreating custom cellular invasion and metastasis models within dynamic complex BBB microenvironment. The 3D high throughput BBB system described here achieves greater reliability in recapitulating the BBB-specific architecture and functions in physiological and pathological processes. Our prototype resembles the *in vivo* BBB microenvironment by incorporating multidimensional aspects such as physical cell-cell interactions, vascular mechanical cues, multiple barrier formation, cell migration and natural 3D ECM. The results obtained by this model are reproducible with the capability to dissect and characterize – not possible by other approaches.

Our study suggests that three key factors must be considered to properly express the barrier phenotypes and functions in endothelial cells of the BBB: physical endothelial-astrocyte interaction, 3D ECM, and dynamic vascular flow. One of the limitations of our approach is the use of rat-derived BMECs and astrocytes to construct the model. It would be ideal to use human derived brain cells in this model, but current difficulty exists in obtaining these cells. We hope that one day we may use of human iPSCs derived brain cells to further recapitulate the BBB in this model system.

In summary, these results reflect the capability of this dynamic organotypic BBB model to represent the functional response of the BBB in pathologies of brain tumor. The model enables the visualization of morphological and/or phenotypic changes of the vascular endothelium and the quantitative evaluation of drug efficacy in tumor cells in parallel, which is not possible with other BBB models *in vitro*. The results suggest that the specific interactions between cancer cells and BBB astrocytes affect the ability of the malignancy to traverse between brain and vascular compartments. The combination of complex physiologically relevant factors capable of replicating endothelial barrier incorporated with the mechanical cues, multicellular architecture and the tumor cells, facilitating the evaluation of drug efficacy in pathologies in a physiologically relevant manner.

## Online Methods

### Cell isolation and cell culture

Primary cultures of rat brain microvascular endothelial cells (BMECs) were prepared from 4-week-old rats (SPF), according to the previously described protocol with our development described as previously published^45^. The rats were killed by cervical dislocation, the bodies were soaked in 75% ethanol for 10 min, the heads were cut off, and then the brains were removed to pre-cooled PBS. The meninges, cerebellum, midbrain and gray matter were carefully separated from the forebrains, and the rest was cleaned to obtain shells of the cortex. The cortices were minced into small pieces of approximately 1 mm^3^, the pieces were digested with 0.1% collagenase type II(Sigma) for 1.5 h at 37 °C, and then the cell pellet was collected by centrifugation (1000 rpm, 8 min, at room temperature). Neural tissues and large vessels were separated from the cell pellet by centrifugation in 20% BSA (Sigma, 4000 rpm, 20 min, 4 °C) to obtain microvascular endothelial cell clusters. The clusters were collected and washed in DMEM (Gibco) twice before plating onto 100-mm plastic dishes (Corning) coated with 0.1 mg/mL collagen type I (BD). The clusters were cultured in ECM (ScienCell) with added bFGF (Gibco, 1 ng/mL) at 37 °C for 24 h, and then the medium was changed every other day until the cultures reached 90–95% confluence. BMECs were passaged by digestion with 0.25% trypsin-EDTA (Gibco) for identification and experiments. BMECs were stained with vWF to identify the characteristic of BMECs, and stained with GFAP to identify the astrocytes as miscellaneous cells. At last, we imaged six random areas of the cells to confirm the characteristics and purity of BMECs.

Primary cultures of cerebral astrocytes were prepared from six neonatal rats of either sex (SPF). The rats were killed by decapitation and, the brains were removed from the skull into a Petri dish containing pre-cooled dissection buffer. The cortex was then carefully separated from the forebrain. Cortical pieces were dissociated mechanically with a suction pipette in DMEM basic medium, filtered (100 μm) and then collected by centrifugation (1500 rpm, 5 min, at room temperature). The cell pellet was resuspended in DMEM basic medium supplemented with 10% FBS (Hyclone) and 1% penicillin- streptomycin solution (Beyotime) and incubated at 37 °C for 50 min. The cell suspension was then placed into six 25 cm^2^ cell culture flasks. The culture medium was changed every 3 days until the cells reached 90–95% confluence. Astrocytes were digested with 0.25% trypsin-EDTA for identification and experiments. Astrocytes were stained with GFAP to identify the characteristics, and dyed with CD11b to identify the microglias as miscellaneous cells. In the end, six random areas were imaged to identify the characteristics and purity of astrocytes.

U87 cells (astrocytoma cell line), A549 cells (lung cancer cell line), MDA-MB-231 cells (breast cancer cell line), M624 cells (melanoma cell line) and BEL-7402 cells (liver cancer cell line) were obtained from the Cell Bank of the Chinese Academy of Sciences (Shanghai, China) and were routinely cultured in DMEM medium supplemented with 10% FBS and 1% penicillin-streptomycin solution at 37 °C in a humidified 5% CO_2_ incubator. U87 cells stably expressing GFP were transfected with a lentiviral vector. A549, MDA-MB-231, M624 and BEL-7402 cells were dyed with DiO (Beyotime) to emit green fluorescence. The culture medium was changed every other day. Upon confluence, the cells were passaged by digestion with 0.25% trypsin-EDTA for experiments. All experiments were performed in accordance with relevant guidelines and regulations, and all experimental protocols were approved by Dalian Institute of Chemical Physics.

### Design of the 3D high throughput BBB microdevice

A microfluidic device was designed for the establishment of a 3D microfluidic BBB system. The chip was composed of 16 independent functional units connected by microchannels and shared the same waste output port in the middle of the chip. Each unit consists of four uniform BBB regions to mimick the BBB *in vivo* one medium channel represents the blood vessel, four gel channels allow gel infusion and gelatin to form the extracellular matrix and one gas channel controls opening and closing of the vascular channel for the loading of medium/cell suspension. The key design of this device was that the height of the vascular channel was almost three times the height of the collagen channel, so that the formation of liquid collagen could be prevented in the collagen channel due to the surface tension caused by the height difference. The 64 blood-brain barrier regions were divided into 16 rows controlled by 16 microvalves, respectively. The microvalves were closed at normal atmospheric pressure and could be opened by evacuating the gas channels with a vacuum pump.

### Fabrication of the integrated BBB microdevice

The microfluidic device used in this work was composed of two PDMS (Dow Corning, USA) layers: the microvalves layer and the cell culture layer, and was fabricated by photolithography. Briefly, SU-8 3035 photoresist (MicroChem, USA) was spin-coated onto clean glass wafers and patterned by photolithography. The master of the microvalves layer was fabricated by coating SU-8 3035 and exposing it to ultraviolet (UV) light once to obtain a thickness of 100 μm. The master of the cell culture layer was fabricated by coating SU-8 3035 and exposing it to UV-light twice, such that the microstructures were of different height. The lower channel was at a height about 70 μm, and the higher channel was at a height about 200 μm, at a width of 400 μm. The PDMS base and curing agents at the ratio of 20:1 by mass for the microvalves layer were mixed together, spin-coated onto the SU-8 masters at 1200 rpm for 30 s, degassed in a vacuum, and cured at 80 °C for 30 min. The PDMS base and curing agent at the ratio of 10:1 by mass for the cell culture layer were mixed together, poured onto the SU-8 master, degassed in a vacuum, and cured at 80 °C for 60 min. Then, the cell culture layer was gently peeled off from the master and bonded to the microvalves layer followed by plasma treatment. After that, the two-layer PDMS replica was gently peeled from the master of the microvalves layer, trimmed to proper size, holes were punched for inlets and outlets, and it was irreversibly bonded to a clean glass wafer after plasma treatment. Prior to the experiments, the device was UV-sterilized for 30 min.

### Establishment of the dynamic blood-brain barriers

Rat tail high-concentration type-I collagen solution was used to mimic the natural extracellular matrix in brain. The collagen solution was compounded at the final concentration of 6mg/mL according to an alternate gelation procedure at 4 °C, aseptically pumped into each collagen channel in the device, and allowed to gel at 37 °C for 30min. Next, the astrocytes suspension at a density of 1 × 10^5^ cells/mL was loaded into the medium channel of the microfluidic device via the cell inlets. The device was turned on its side for 10 min to allow the astrocytes to adhere to the surface of the collagen gel, and then BMECs were loaded into the device using the same method. After incubation in a humidified incubator at 37 °C for 48 h, the astrocytes and BMECs cultured on the surface of the collagen gel distributed and proliferated together to form a BBB *in vitro*. BMECs mono-cultured in the device were used as the control group, and the experiments were performed under both static and flow conditions described below. To monitor the morphology of the BBB, the astrocytes and BMECs were individually dyed with Hoechst 33258 (blue, Beyotime) and DiI (red, Beyotime) fluorescent dye, respectively, before loading.

### Generation of fluidic flow and control

Extremely low fluidic flow was applied to the medium channels to mimic the blood flow in the microvessels as described previously^31^,^46^. The microdevice was perfused at a flow velocity at about 20 μL/min following cell seeding, and then the flow velocity in the medium channel was decreased to ~1 μL/min due to the split flow of the chip. We used an approximation formula to calculate the flow shear stress (FSS) as:


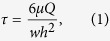


where τ is the FSS to the BMECs, μ is the dynamic viscosity of the medium (0.012 dyne·s/cm^2^), Q is the flow velocity (1 μL/min), and w and h are the width (400 μm) and height (200 μm), respectively, of the medium channel. According to formula 1, the FSS in this assay was about 0.1 dyne/cm^2^.

### Immunofluorescence staining and imaging

Cell samples were washed three times in PBS before fixing in 4% paraformaldehyde (Sigma) for 30 min and permeabilized in 0.2% Triton X-100 solution (Sigma) for 15 min. The samples were then blocked in blocking serum (Solarbio) and incubated with VE-Cadherin (Santa Cruz), ZO-1 (Santa Cruz), Claunin-5 (Proteintech), P-glycoprotein (Cell Signaling), Glut-1 (Proteintech), respectively diluted in primary antibody dilution buffer (Solarbio) at 4 °C for 24 h. Target proteins of the samples were detected by incubating cells in fluorophore-conjugated secondary antibodies (Zhongshan Golden Bridge) for 1 h at room temperature away from light. Cell nuclei were counterstained with DAPI (Sigma). Images were obtained with an Olympus IX71 fluorescence microscope (Olympus Corporation) equipped with a CCD digital camera (Leica) and processed with Leica Application Suite (Leica) and Image-Pro Plus software (Media Cybernetics, USA). Fluorescence images of single BBB region were taken using a Laser Scanning Confocal Microscope (Olympus Fluo View TM FV1000).

### Molecular permeability assay

To assess the permeability of the endothelial barrier against small compounds, fluxes of fluorescent molecules were measured under both static and flow conditions by detecting the fluorescence intensity of sodium fluorescein (NaFl, 376Da, Sigma) diffusion across the BBB. The initial concentration of NaFl was 250 μM and was delivered into the medium channel after the formation of the barrier. Time-course images of the NaFl on the brain side were then collected. The absolute intensities of NaFl penetrating the barrier were measured to evaluate the permeability of BBB by taking fluorescent images in brain side at different time points. The fluorescent intensity of NaFl of three random areas on the brain side was measured and quantified using Image-Pro Plus software (Media Cybernetics, USA).

### Transendothelial electrical resistance (TEER) measurement

To evaluate the integrity and barrier function of the 3D high throughput BBB system, the TEER value was determined to monitor the formation of the BBB according to the methods recorded previously, with minor modification^23^,^47^. A resistance meter was used to measure the TEER. The positive electrode was put in the cell inlet, the negative electrode was put in the collagen inlet, and the resistance value (R) was read every 4 h for 80 h, and each time point was read three times. The initial background resistance of the collagen (R_0_) was tested in the same manner. The cell culture area of the BBB (A) was 1.6 × 10^−3^ cm^2^, and the TEER value in Ω × cm^2^ was calculated as:


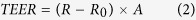


### Permeability test of pharmaceutical agents crossing the BBB

Eight different chemotherapeutic drugs in clinical use were prepared for this assay. As reported previously^37^, only temozolomide (TMZ, Santa Cruz) is lipid soluble and can pass through the BBB into the brain side *in vivo*. The other seven chemotherapeutic drugs are water soluble and are normally used clinically to treat many other tumors but not brain tumors. Given its solubility, TMZ was dissolved in DMSO, and the other drugs were dissolved in PBS. All eight drugs were prepared at a concentration twice that of their respective IC_50_ values^37^,^38^,^39^,^40^,^41^,^42^,^43^,^44^. Three groups of experiments were performed: control group (no barrier), BMECs mono-cultured group and BBB group (BMECs co-cultured with astrocytes). Drug solutions were individually pumped into the medium channels, and all cells were dyed with Live/Dead kits (Molecular Probes) according to the protocol after 48 h. Images were obtained by fluorescence microscopy, and fluorescence intensity was calculated by Image-Pro Plus software (Media Cybernetics, Rockville, MD) for evaluation of cell apoptosis.

### Transendothelial migration assay

The transendothelial migration assay of cancer cells crossing the BBB was performed as previously described with minor modification^48^. Prior to assay, the astrocytes and BMECs were stained with Hoechst 33258 and DiI live staining dyes, respectively. The different cancer cells were digested and resuspended to the density of 5 × 10^4^ cells/mL, loaded into the medium channel, and adhered to the BBB by turning the device on its side for 5 min, as done to originally establish the BBB, and then the device was incubated in a humidified incubator at 37 °C. Tumor cells were allowed to invade for 72 h, and images were collected every 24 h by a fluorescence microscope equipped with a CCD digital camera. For the invasion assay of endogenous U87 brain tumor cells, the cells were digested and resuspended to the density of 1 × 10^6^ cells/mL and pre-dyed with DiO (green, Beyotime) prior to use. Before establishing the BBB, the U87 cells were mixed together with collagen solution and loaded into the collagen channels. After gelling, the U87 cells were embedded in the collagen gel in a 3D microenvironment to mimic the brain tumor *in vivo*. The 3D high throughput BBB system was subsequently established, and images were obtained every 24 h beginning 48 h after the BBB was formed. All of the invasion assays were performed under flow conditions.

### Interaction assay of astrocytes and tumor cells

To investigate the interaction between the astrocytes and tumor cells, astrocytes were individually co-cultured with the five kinds of tumor cells (U87, A549, MDA-MB-231, M624 and BEL-7402 cells). Prior the assay, the astrocytes were pre-dyed with DiI, and tumor cells were pre-dyed with DiO. All cell types were prepared at a density of 5 × 10^5^ cells/mL and added to a Petri dish at a ratio of 1:1 by volume. Images were collected 48h after co-culture.

## Additional Information

**How to cite this article**: Xu, H. *et al*. A dynamic *in vivo*-like organotypic blood-brain barrier model to probe metastatic brain tumors. *Sci. Rep*. **6**, 36670; doi: 10.1038/srep36670 (2016).

**Publisher’s note:** Springer Nature remains neutral with regard to jurisdictional claims in published maps and institutional affiliations.

## Supplementary Material

Supplementary Information
