# Supplementary material for: A dynamic in vivo-like organotypic blood-brain barrier model to probe metastatic brain tumors
Source: Sci Rep. 2016 Nov 10;6:36670. doi: 10.1038/srep36670 (PMC5103210; doi:10.1038/srep36670)
Supplement: Supplementary Information [file srep36670-s1.pdf]

A dynamic in vivo-like organotypic blood-brain barrier model to probe metastatic brain tumors

Hui Xu[1,2], Zhongyu Li[1,2], Yue Yu[1,2], Saman Sizdahkhani[3], Winson S. Ho[3], Fangchao Yin[1,2], Li Wang[1], Guoli Zhu[1,2], Min Zhang[1], Lei Jiang[1], Zhengping Zhuang[3], and Jianhua Qin[1,*]

[1]Division of Biotechnology, Dalian Institute of Chemical Physics, Chinese Academy of Sciences, Dalian, China. [2]University of Chinese Academy of Sciences, Beijing, China. [3]Surgical Neurology Branch, National Institute of Neurological Disorders and Stroke, National Institutes of Health, Bethesda, Maryland, USA.

Correspondence should be addressed to J.H.Q. (jhqin@dicp.ac.cn).

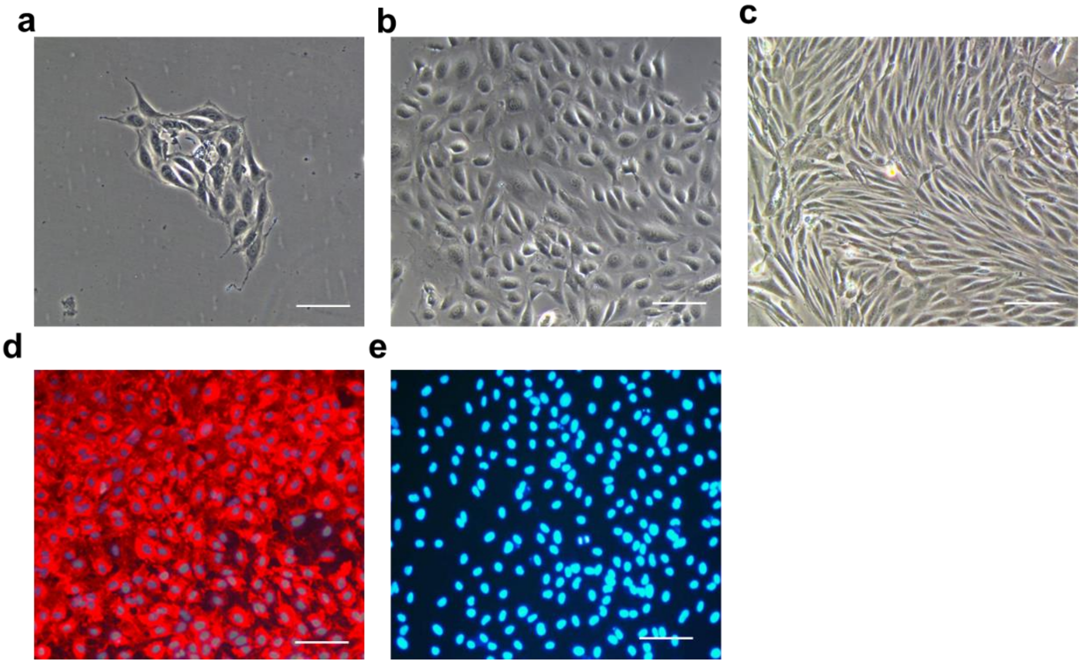

Supplementary Figure 1 Extraction and characterization of primary BMECs. (**a-c**) Phase contrast images of primary BMECs after extraction of 3 days (**a**), 7 days (**b**) and 10 days (**c**). (**d-e**) Fluorescent images of the purified primary BMECs expressing cell-specific markers. (**d**) Expression of vWF (red, endothelial cell marker), and (**e**) expression of GFAP (green, astrocyte marker). DAPI (blue) is shown overlaid with vWF and GFAP. All scale bars indicate 50 µm.

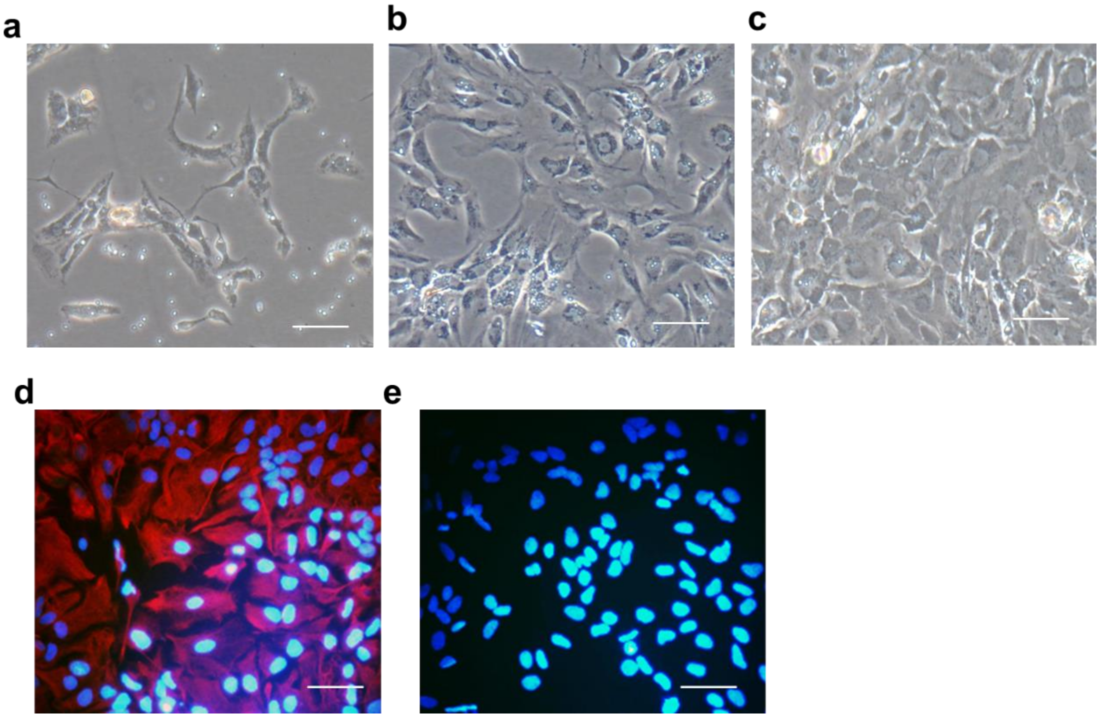

Supplementary Figure 2 Extraction and characterization of primary brain astrocytes. (**a-c**) Phase contrast images of primary brain astrocytes after extraction of 3 days (**a**), 6 days (**b**) and 9 days (**c**). (**d-e**) Fluorescent images of the characteristic cell markers expressed by purified astrocytes. (**d**) Expression of GFAP (red, astrocytes marker), and (**e**) expression of CD11b (green, microglia marker). DAPI (blue) is shown overlaid with GFAP and CD11b. All scale bars indicate 50 μm.

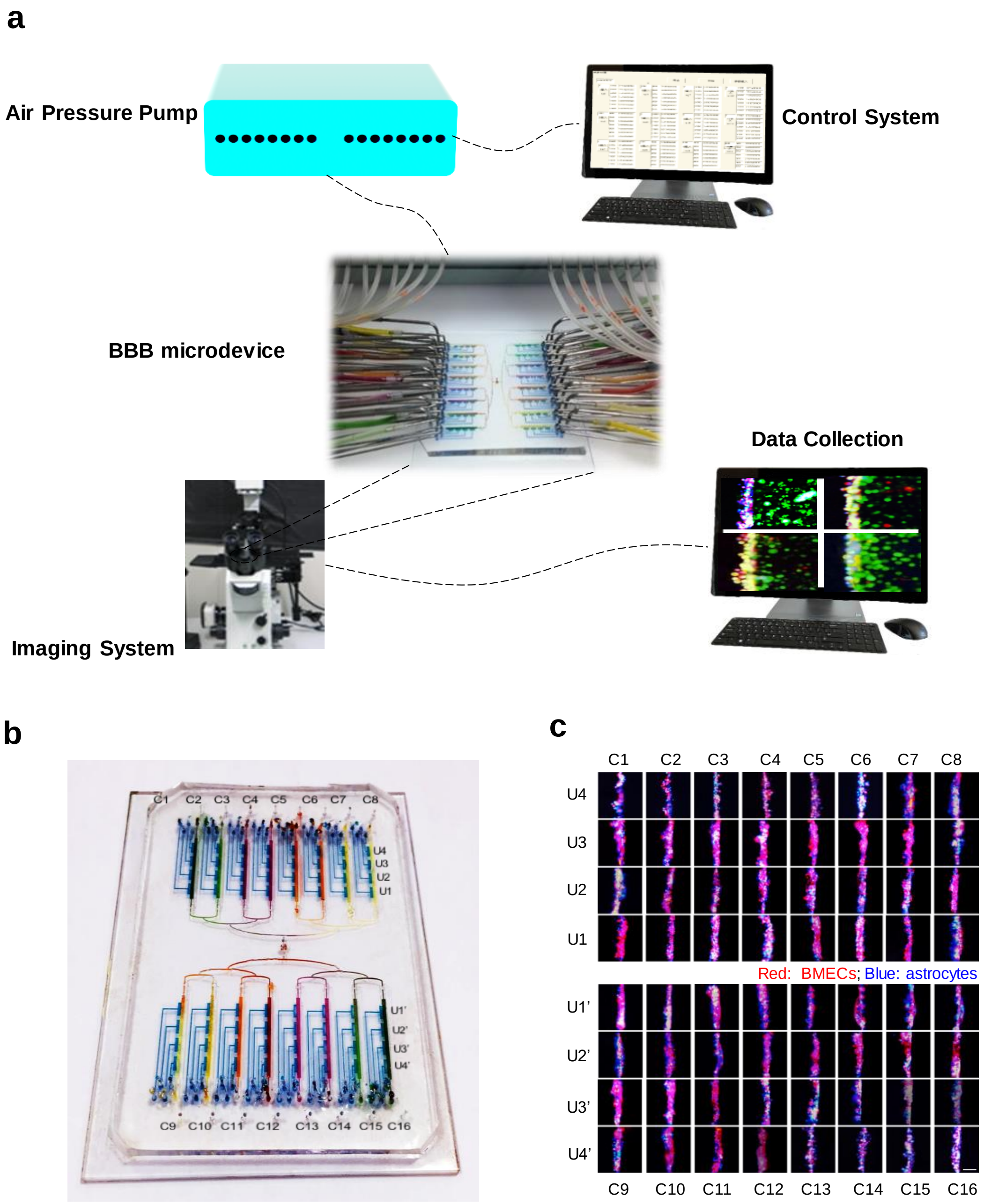

Supplementary Figure 3 Configuration of the high throughput BBB system. (**a**) The major components of the system are BBB microdevice, air pressure pump, control system, imaging and data collection system. (**b**) Photograph of the actual microdevice. (**c**) Fluorescent images of 64 blood-brain interfaces formed on the 3D high throughput BBB system simultaneously.

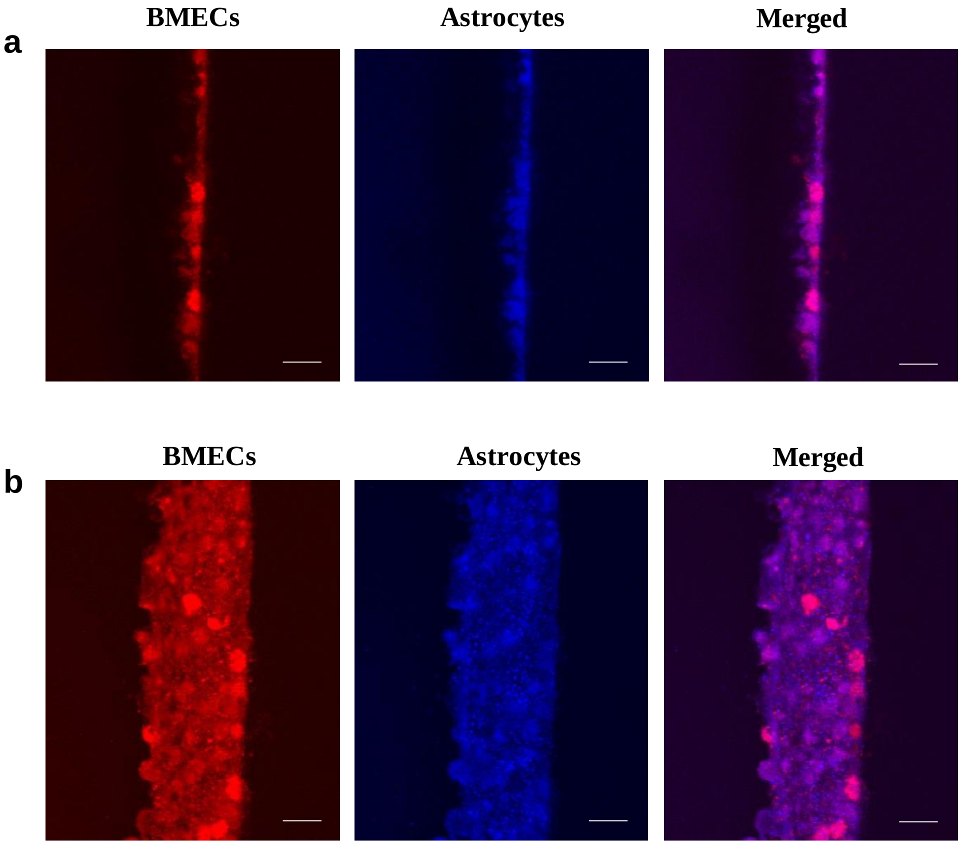

Supplementary Figure 4 Confocal images of blood-brain barrier. (a) Cross-section of the blood-brain barrier. (b) Front-view of the blood-brain barrier. Red: BMECs; Blue, astrocytes. Scale bar, 50μm.

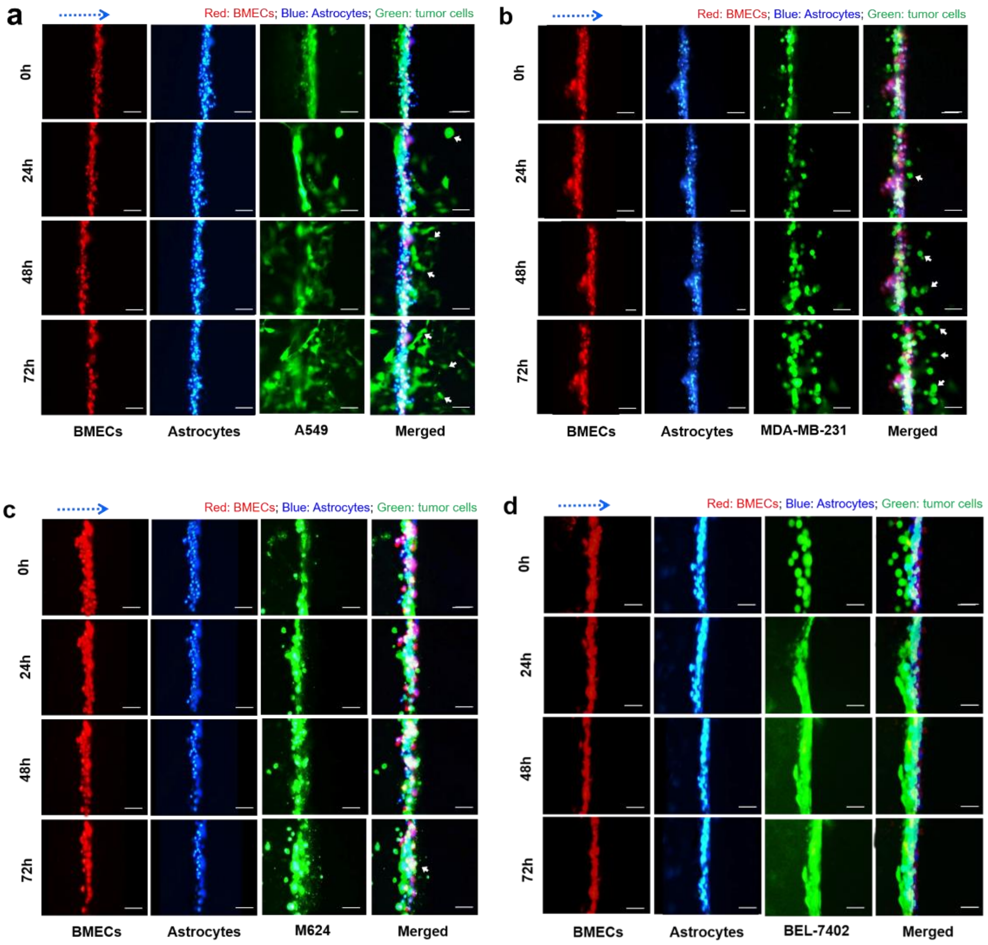

Supplementary Figure 5 Time-lapse images of extravasation of different cancer cells across the barrier on this BBB system. (**a**) lung cancer cells (A549 cells), (**b**) breast cancer cells (MDA-MB-231 cells), (**c**) melanoma cells (M624 cells), and (**d**) liver cancer cells (BEL-7402 cells). Red, BMECs; Blue, astrocytes; Green, U87 cells. All scale bars indicate 100μm.

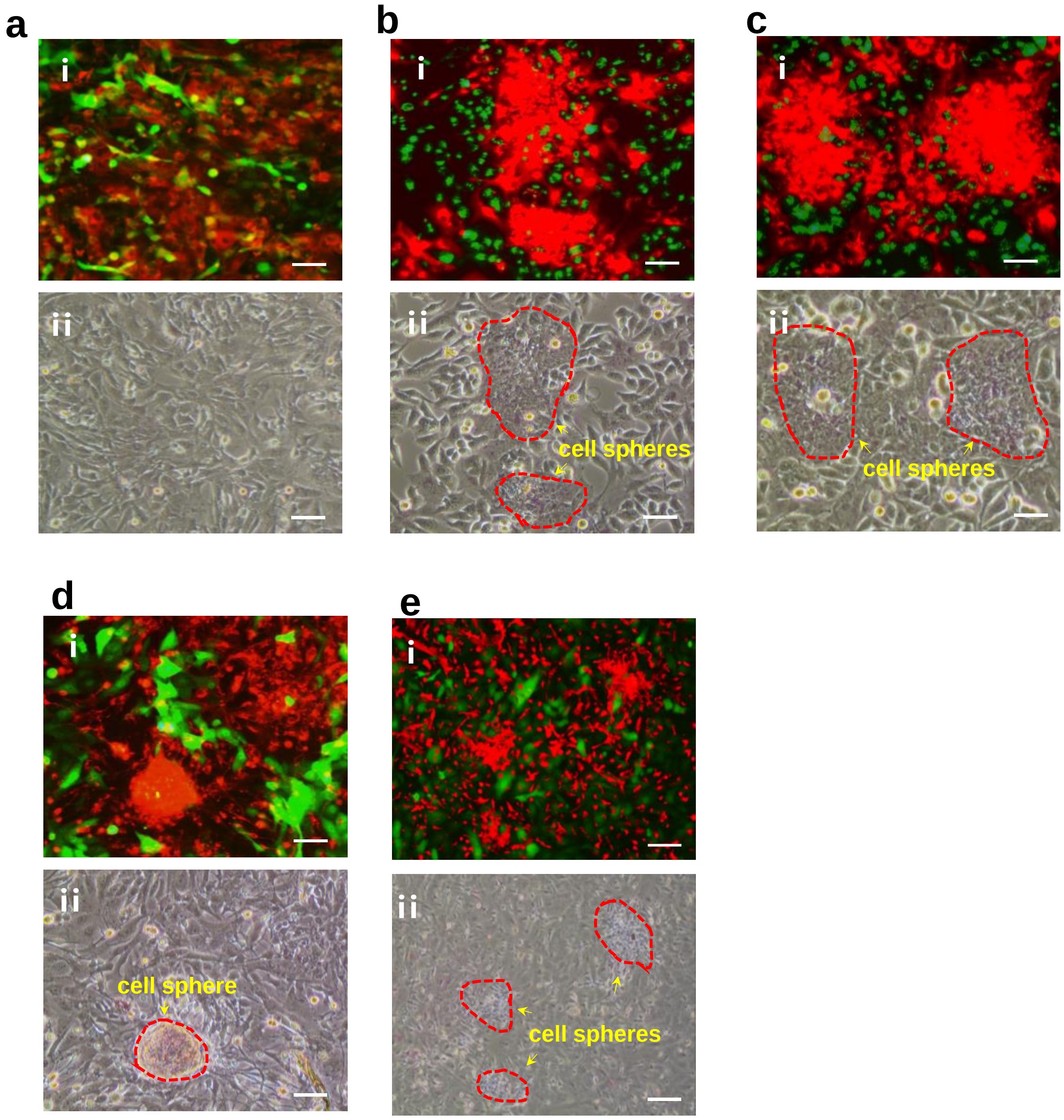

Supplementary Figure 6 Interaction assay between astrocytes and different cancer cells. Co-culture of astrocytes (red) with (**a**) brain cancer cells (U87 cells, green), (**b**) lung cancer cells (A549 cells, green), (**c**) breast cancer cells (MDA-MB-231 cells, green), (**d**) melanoma cells (M624 cells, green), and (**e**) liver cancer cells (BEL-7402 cells, green). Fluorescent images are shown in (**i**), and phase contrast images are shown in (**ii**). Circles: clusters of cell spheres. All scale bars indicate 50μm.

Supplementary Table 1 Clinical drug compounds tested in this study

Compounds	Structural formula	Molecular weight (Da)	Solubility	Permeability through BBB	Clinical application	Mechanism of action: Induction of apoptosis	Mechanism of action: Hepatic metabolism	IC_{50}
Temozolomide (TMZ)	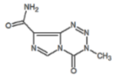	194.20	Lipophilic	√	Glioblastoma	√	X	200 μM[37]
Carboplatin (CBP)	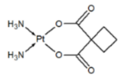	371.26	Hydrophilic	X	Ovarian cancer, lung cancer, esophageal cancer	√	X	100 μM[38]
Cisplatin (DDP)	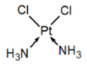	300.05	Hydrophilic	X	Ovarian cancer, carcinoma of testis	√	X	50 μM[39]
5-Fluorouracil (5-Fu)	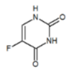	130.08	Hydrophilic	X	Breast cancer, cervical carcinoma	√	X	3 μM[40]
Nedaplatin (NDP)	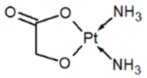	303.18	Hydrophilic	X	Lung cancer, ovarian cancer, ovarian cancer,	√	X	100 μM[41]
Gemcitabine (GEM)	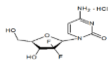	299.66	Hydrophilic	X	Lung cancer, pancreatic cancer	√	X	500 nM[42]
Tegafur (FTO)	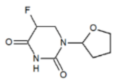	200.17	Hydrophilic	X	Gastric cancer, intestinal cancer, liver cancer	X	√	200 μM[43]
Ifosfamide (IFO)	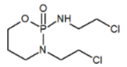	261.10	Hydrophilic	X	Breast cancer, liver cancer	X	√	300 μM[44]

BBB, blood-brain barrier; IC_{50}, half maximal inhibitory concentration.

Supplementary Table 2 Parameters of BBB models and the 3D high throughput (HT) BBB system

System type	Animal model	Transwell BBB	DIV-BBB	Sandwich chip	3D HT BBB
Citations	8-9	10-16	17-22	23-31	
Physical cell-cell contact	+	±	-	±	+
ECM	+	±	-	±	+
Dynamic flow	+	-	+	±	+
TEER ($\Omega \times cm^2$)	1500-2000	60-1400	500-1200	30-300	~1300
Time to steady-state TEER	-	3-4days	9-12 days	3-4 days	2-3days
Cell migration	-	-	-	-	+
Visualization of time-resolved barrier	±	-	-	-	+
High throughput	-	-	-	+	+
Repeatability and controllability	-	+	+	+	+

BBB, blood-brain barrier; DIV, dynamic in vitro; ECM, extracellular matrix; TEER, transendothelial electrical resistance.
